# Isolation of a novel alginate lyase‐producing *Bacillus litoralis* strain and its potential to ferment *Sargassum horneri* for biofertilizer

**DOI:** 10.1002/mbo3.387

**Published:** 2016-07-20

**Authors:** Mingpeng Wang, Lei Chen, Zhengyi Liu, Zhaojie Zhang, Song Qin, Peisheng Yan

**Affiliations:** ^1^ School of Municipal and Environmental EngineeringHarbin Institute of TechnologyHarbinChina; ^2^ Yantai Institute of Costal Zone Research Chinese Academy of SciencesYantaiChina; ^3^ Department of Zoology and PhysiologyUniversity of WyomingLaramieWyomingUSA; ^4^ School of Marine Science and TechnologyHarbin Institute of TechnologyWeihaiChina

**Keywords:** alginate oligosaccharide, *Bacillus litoralis*, biofertilizer, fermentation, *Sargassum horneri*

## Abstract

Algae have long been used to augment plant productivity through their beneficial effects. Alginate oligosaccharide is believed to be one of the important components to enhance growth and crop yield. In this study, we isolated and characterized a *Bacillus litoralis* strain, named *Bacillus* M3, from decayed kelps. We further demonstrated that the M3 strain could secrete alginate lyase to degrade alginate. The crude enzyme exhibited the highest activity (33.74 U/mg) at pH 7.0 and 50°C. The M3 strain was also able to ferment the brown alga *Sargassum horneri*. Fermentation results revealed that a fermentation period of 8–12 hr was the best harvest time with the highest level of alginate oligosaccharides. Plant growth assay showed that the seaweed fermentation extract had an obvious promotion effect on root and seedling growth of *Lycopersicon eseulentum* L. Our results suggest that fermentation extract of *Sargassum horneri* by the novel strain of *Bacillus litoralis* M3 has significant development potential for biofertilizer production and agriculture application.

## 1 Introduction

As sources of organic matter and fertilizer, seaweeds have been used as soil conditioners and fertilizers for centuries (Blunden, 1972; Temple & Bomke, 1988). Brown seaweeds are most commonly used in agriculture (Ugarte, Sharp, & Moore, 2006). A number of commercial products extracted from seaweeds are available for use in agriculture and horticulture. Most manufacturers use the brown alga *Ascophyllum nodosum*as raw material to produce seaweed fertilizer (Khan et al., 2009).

At present, numerous studies have revealed a wide range of beneficial effects of seaweed extract applications on plants, such as early seed germination and establishment, improved crop performance and yield, elevated resistance to biotic and abiotic stress, and enhanced postharvest shelf‐life of perishable products (Bai, Christi, & Kala, 2013; Kaoaua, Chernane, Benaliat, & Neamallah, 2013; Mohamed & El‐Sehrawy, 2013). Seaweed components such as alginate, amino acids, vitamins, and plant hormones affect cellular metabolism in treated plants leading to enhanced growth and crop yield (Craigie, 2010; Durand, Briand, & Meyer, 2003; Lane, Mayes, Druehl, & Saunders, 2006).

Alginate is an acidic linear polysaccharide distributed widely in the cell walls of brown algae. Alginate consists of hexuronic acid residues β‐d‐mannuronic acid (M) and α‐l‐guluronic acid (G) with exclusively 1→4 glycosidic linkages and can be degraded into alginate oligosaccharides with low degree of polymerization by alginate lyases (Gacesa, 1988; Ji, 1997; Preiss & Ashwell, 1962). Alginate oligosaccharides have been reported to have beneficial effects on different plants, such as promoting the growth of banana plantlets, root growth‐promoting activity toward lettuce, and alleviating salt stress for *Brassica campestris* L. (Iwasaki & Matsubara, 2000; Natsume, Kamo, Hirayama, & Adachi, 1994; Tang, Zhou, Chu, & Nagata, 2011; Yonemoto et al., 1993). Recently, a series of studies have partly revealed some functional mechanism for the action of alginate oligosaccharide (Liu et al., 2013; Zhang et al., 2014). Their results suggested that this marine oligosaccharide could be widely used for crop development.

Although many of the various chemical components of seaweed extracts were demonstrated beneficial to plants, it is difficult to extract all these components at the same time. The cross‐linked structure of alginate and its high concentration in cell wall were a big obstacle for extracting effective components from brown seaweed cells (Ji, 1997; Michel, Tonon, Scornet, Cock, & Kloareg, 2010). The key process in seaweed fertilizer production is to effectively break the seaweed cell wall to extract active components and simultaneously keep their activities.

Fermentation is a metabolic process involving substance conversion that occurs in some microorganisms. It has been used for the production of food, beverages, drugs, biofuel, and other uses (Clarens, Nassau, Resurreccion, White, & Colosi, 2011; Ekanayake et al., 2008; Gupta, Abu‐Ghannam, & Scannell, 2011). A stable and effective microbe is the foundation of fermentation. For example, *Saccharomyces cerevisiae* for beer production and *Lactobacillus bulgaricus* for yogurt production. An alginate‐degrading strain might be crucial for cell wall breakdown and seaweed fermentation. In searching for good candidates of alginate‐degrading microbes, we found some decayed kelps stored for a long period of time in our laboratory refrigerator. Preliminary screening revealed that the decay was likely caused by microbes growing on the kelps.

In this study, we identified an alginate‐degrading strain from the decayed kelp and tested for fermentation of *Sargassum horneri* extracts. As an important nutrient derived from seaweed, alginate oligosaccharide was detected in fermentation broth for the first time by thin layer chromatography. The seaweed fermentation extract was applied to test the enhancement effect on root and seedling growth of tomato.

## 2 Materials and Methods

### 2.1 Preparation of seaweed water extract

The brown alga *Sargassum horneri* was collected during low tide periods, from the coastal area of Rongcheng, located in the eastern tip of Shandong Peninsula. Seaweed was washed with tap water to remove shell, debris and sand. Samples were air‐dried (26°C) in 2–3 days followed by thermostat drying at 60°C for 48 hr. Dried seaweeds were hand crushed and powdered with high‐speed universal grinder. Algae powder was mixed with sterile distilled water in a ratio of 1:20 (w/v) and autoclaved at 121°C for 20 min. After cooling, the seaweed broth was centrifuged at 15,280 g for 10 min. The supernatant was then collected in a sterile condition as seaweed water extract (SWE) and stored at 4°C for further experimental studies.

### 2.2 Isolation and screening of microorganism for seaweed fermentation

The kelps of *Laminaria japonica* were collected from Nanhuangcheng Island of Shandong province, China (38°21′N; 120°54′E) and kept at 4°C in our laboratory. We found some of the decayed kelps were covered with various bacterial consortia. For isolation of fermentative microorganism, bacteria were scraped and diluted successively with sterilized water and then spread on seaweed extract agar (SWE with 2% agar). After 24–48 hr of incubation at 30°C, morphologically different colonies were selected and further purified by repeatedly streaking on the same medium. The purified strains were inoculated into SWE medium, collected and stored in 30% glycerol at −80°C.

The purified isolates were inoculated in liquid alginate (ALG) medium, consisting of 0.5% (w/v) sodium alginate, 0.5% (NH_4_)_2_SO_4_, 0.2% KH_2_PO_4_, 0.1% MgSO_4_·7H_2_O, and 0.002% FeSO_4_·7H_2_O (pH 7.2) for secondary screening. After being incubated for 24 hr at 30°C with shaking (200 rpm), cells were centrifuged at 15,280 g, 4°C, for 5 min. The supernatants were collected to determine the alginate‐degrading and alginate lyase activity.

### 2.3 Identification and characterization of the microorganism

Phenotype characteristics of the microorganisms, including gram reaction, cell shape, motility, and sporulation were determined as described in Bergey's Manual of Determinative Bacteriology (Bergey & Holt, 1994). Morphology of the microorganisms was examined using an optical microscope (Olympus BX51). Cells were placed on a microscope slide, then observed directly with a 100× objective. Pictures were taken using Olympus DP72 camera and Image‐Pro Plus 5.1 software. Acid production from D‐Mannitol, a key character of *Bacillus litoralis*, was determined according to the method as described by Leifson (Leifson, 1963).

Genomic DNA from selected bacterial cultures was extracted as described by Mora (Mora, Fortina, Parini, Daffonchio, & Manachini, 2000). The 16S rRNA gene was amplified using primers 27F (5′‐AGAGTTTGATCCTGGCTCAG‐3′) and 1492R (5′‐TACGGTTACCTTGTTACGACTT‐3′) (Weisburg, Barns, Pelletier, & Lane, 1991). The sequencing of the purified PCR product was performed by Invitrogen Ltd. The taxonomical identification of microbe was performed by BLAST search (http://blast.ncbi.nlm.nih.gov/Blast.cgi). Phylogenetic tree was constructed using neighbor‐joining method with bootstrap (1,000 replicates) by Kimura 2‐parameter model using MEGA 5.1 program (Kumar, Nei, Dudley, & Tamura, 2008).

### 2.4 Measurement of soluble alginate content

The soluble alginate content was measured using sodium diethyldithiocarbamateas described by Wang (Wang, Leng, Xing, & Zhai, 2009). Briefly, 200 μl copper sulfate solution (10 mg/ml) was added into 2 ml supernatant of fermentation broth and the supernatants were collected by centrifugation at 15,280 g for 5 min. The precipitate was washed with distilled water for three times. All the fractions of supernatants were mixed in a 50 ml volumetric flask by adding distilled water to the volume 50 ml. 1.5 ml of this solution was transferred into a new 50 ml volumetric flask. The pH was adjusted to 8.5 via adding 1 ml EDTA (2% w/v)‐ammonium citrate (4% w/v) buffer and 3 ml ammonia (4.8% v/v)‐ammonium chloride (7% w/v) buffer. 5 ml of sodium diethyldithiocarbamate (2 mg/ml) was added. Finally, distilled water was added to the volume of 50 ml and the absorbance measured at 447 nm using a TU‐1901 spectrometer (Persee, Beijing). The content of excessive copper ion was determined by the standard curve. The soluble alginate content was calculated by conversion factor 6.24 according to the chemical equilibrium (Haug & Smidsrød, 1965).

### 2.5 Assay of alginate lyase activity

After 24 hr incubation in ALG medium, 300 μl of supernatants of M3 fermentation broth were used to determine the initial alginate lyase activity. 200 ml supernatants were precipitated by ammonium sulfate (80% saturation) and stored at 4°C overnight. The crude enzyme proteins were collected by centrifugation at 15,280 g for 15 min, and then dissolved in 1 ml PBS buffer and 0.3 ml of this solution were mixed with 2.7 ml Tris‐HCl (50 mM, pH 7.0) containing 0.5% sodium alginate (Sinopharm Chemical Reagent Co., Shanghai, China). The mixture was incubated at 50°C for 10 min. The reaction was stopped by heating in boiling water for 10 min. The enzyme activity was then assayed by measuring the increased absorbance at 235 nm. One unit was defined as the amount of enzyme required to increase the absorbance at 235 nm by 0.1 per min. The protein concentrations were determined with a protein quantitative analysis kit (TransGen Biotech, Beijing, China).

### 2.6 Characterization of the crude alginate enzyme

The pH effect on crude alginate lyase activity was determined by incubating the enzyme in 50 mM Tris–HCl buffer with the pH range from 4 to 10 at an interval of 0.5‐pH unit at 30°C for 10 min. The effect of temperature on enzymatic activity was determined at temperatures ranging from 25 to 60°C for 10 min in 50 mM Tris–HCl buffer (pH 7.0). Substrate specificity of crude alginate lyase was investigated using 0.5% (w/v) poly β‐D‐mannuronate (polyM) and poly α‐l‐guluronate (polyG)(Qingdao BZ Oligo Biotech Co., Qingdao, China), respectively. The assay of enzyme activity was defined as described above.

### 2.7 Shake‐flask fermentation

Three media were used for shake‐flask fermentation in our study. In addition to SWE and ALG media described above, ALG + SWE medium (seaweed water extract with 0.25% sodium alginate) was also used. A purified single colony was inoculated into 5 ml marine broth 2216 (Difco) for overnight. Then 200 μl of overnight culture was transferred into 20 ml marine broth 2216 for 12–16 hr and used as starter culture. Starter culture was inoculated into 100 ml of three different media with 1% and 10% (v/v) inocula and incubated at 30°C with shaking at 200 rpm for 24 hr to carry out fermentation. A 3 ml aliquot was drawn at interval of 4 hr and analyzed for cell density, pH, soluble alginate content, and alginate oligosaccharides.

### 2.8 Fermentation in 30 L bioreactor

2 kg seaweed powder was mixed with 20 L distilled water (1:10 w/v) in a 30 L bioreactor (GAO JI bio‐engineering Co., Ltd, Shanghai) and autoclaved at 121°C for 20 min. After cooling, 10% starter culture was inoculated into the mixture. Fermentation started with controlling pH as required at 30°C and 200 rpm. The pH was controlled at 7.0 via adding ammonia automatically according to on‐line monitoring with pH electrode. Samples at every four hours were analyzed as described above. Fermentation broth at 12 hr and 24 hr was collected at and centrifuged to remove bacteria cells as seaweed fermentation extract (SFE‐12 and SFE‐24, respectively), which was stored at 4°C for agriculture test.

### 2.9 Thin layer chromatography

Alginate oligosaccharides in fermentation broth were detected by thin layer chromatography (TLC). Aliquot samples were taken at the interval of 4 hr and spotted on a silica gel high‐performance TLC plate (Merck, Germany). Alginate oligosaccharides were developed with a solvent system of 1‐butanol‐formic acid‐water, (4:6:1 v:v:v) (Kim, Lee, & Kim, 2009). The developed plate was stained by heating the TLC plate at 110°C for 5 min after spraying with 10% (v/v) sulfuric acid in ethanol. The mannuronic acid sodium salt dimer, trimer, tetramer, and heptamer (1.25 mg/ml) (Qingdao BZ Oligo Biotech Co., Qingdao, China) were used as standards.

### 2.10 HPLC analysis of alginate oligosaccharides

The crude enzyme (0.5 ml) was added into 50 ml Tris–HCl (50 mM, pH 7.0) containing 0.5% sodium alginate and incubated at 50°C for 24 hr. The reaction was stopped by heating in boiling water for 10 min. Then samples from this enzymatic hydrolysate and seaweed fermentation broth (SFE‐12) were treated by Sevag method to remove proteins (Staub, 1965) and filtered with 0.22 μm filter. Chromatographic analysis of alginate oligosaccharides was performed on UltiMate 3000 system equipped with a PDA detector (Waters 2996, Waters, USA). A Superdex 30 column (10 mm × 300 mm, GE Healthcare, USA) maintained at 25°C was used as the separation channel. Isocratic elution was performed at 25°C with mobile phase consisting of 0.1 mol/L NaCl at a constant flow rate of 0.2 ml/min. The injection volume for standards and samples were 20 μl. UV detection was carried out at 230 nm. The mannuronic acid sodium salt dimer, trimer, and tetramer (5 mg/ml) were used as standards.

### 2.11 Analysis of plant growth treated with different seaweed extract

The seeds of tomato (*Lycopersicon eseulentum* L.) were purchased from market (Agricultural Science and Technology Garden, Yantai) and were treated with different test solutions. First of all, alginate oligosaccharides in enzymatic hydrolysate (as described above) were diluted to the same concentration in SFE‐12 and this diluted solution was defined as alginate oligosaccharides solution (AOS). Seaweed water extract (SWE) and seaweed fermentation extract (SFE‐12 and SFE‐24) were diluted in two concentrations of 0.25% and 1% with distilled water. As a positive control, AOS were diluted to 0.25% and 1% and a commercial seaweed fertilizer (LEILI, Beijing; defined as CSF) was diluted according to the instructions. Distilled water was used as a negative control.

For plant growth, Dry tomato seeds (60 seeds per treatment) were soaked in these test solutions for 12 hr and then washed three times with distilled water. Thirty similar seeds of every treatment were selected and divided into batch of 10 each. Seeds were arranged in pots (10 cm diameter) at a depth of 2.5 cm below the soil level and allowed to germinate. After germination in each pot five healthy plants were retained and other plants were removed. The length of the main root and the seedling was measured after 7 days incubation. The results were expressed as percentage of the control.

### 2.12 Strain deposition and sequence accession

The M3 strain has been deposited in the China Center for Type Culture Collection under the accession number CCTCC AB2015440. The accession number for the 16S rRNA gene sequence of M3 strain was KU360237.

## 3 Results

### 3.1 Screening and characterization of microorganism

**Figure 1:**
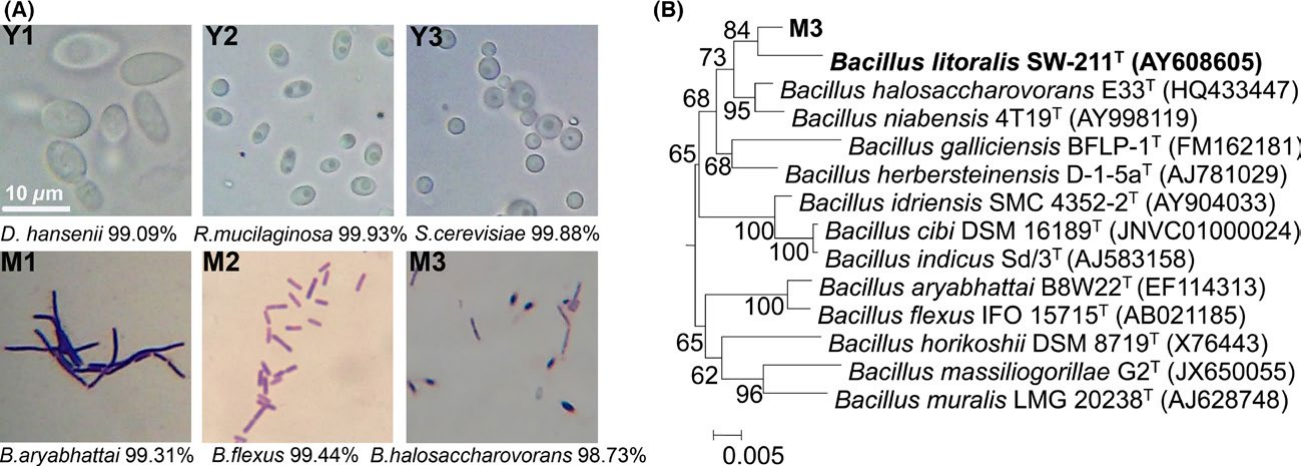
Isolation and identification of fermentative strains. (A) Phenotypic characterization of selected cultures. Microorganism mixture was spread on seaweed water extract (SWE) plate and incubated at 30°C for 48 hr. Six morphologically different colonies were selected for microscopy and gram reaction. All strains were identified by 16S or 18S rRNA sequencing and the most closely phylogenetically related species of each strain were also listed. (B) Phylogenetic analysis of M3 strain. Phylogenetic tree was constructed using neighbor‐joining method with bootstrap (1000 replicates) by Kimura 2‐parameter model using MEGA 5.1 program

Bacteria were scraped from surface of decayed kelp and serially diluted to spread on seaweed extract agar plates. Six different kinds of predominant colonies were selected and examined for gram reaction, cell shape, motility, and sporulation. By morphological observation, these strains were identified as three yeasts (Y1‐Y3) and three bacteria (M1‐M3) (Fig. 1A). All three bacteria were gram positive and able to sporulate. The M2 and M3 strains were motile. These selected cultures (6 isolates) were identified by 16S rRNA sequencing. The sequences containing at least 1,300 bp were used for database query. Sequence BLAST using MEGABLAST tool of GenBank revealed that the three yeast isolates were *Debaryomyces hansenii*;* Rhodotorula mucilaginosa,* and *Saccharomyces cerevisiae*; the three bacterial isolates were *Bacillus aryabhattai*;* Lysinibacillus xylanilyticus,* and *Bacillus halosaccharovorans*. These selected cultures were further analyzed for their ability to grow in SWE and ALG media. All of them grew well in SWE. But only M3 strain could grow in ALG and degrade alginate (Fig. S1). This strain was identified as alginate‐degrading and selected for further study.

To further determine the taxonomic position of M3 strain, more tests and analysis were applied (Table 1). Phylogenetic analysis of the 16S rRNA gene sequence of M3 strain (1,443 bp) indicated that its closest relatives were *Bacillus halosaccharovorans* E33^T^ (98.73% identity) and *Bacillus litoralis* SW‐211^T^ (98.29% identity). The neighbor‐joining tree (Fig. 1B) revealed that M3 strain clustered most closely with *Bacillus litoralis*. M3 strain was therefore named as *Bacillus litoralis* M3.

**Table 1:** Characteristics that distinguish strain M3 from members of the most closely phylogenetically related species of the genus *Bacillus*

Characteristic	M3	*Bacillus litoralis* aSW‐211^T^	*Bacillus halosaccharovorans* bE33^T^	*Bacillus niabensis* cIBRC‐M10590^T^	*Bacillus herbersteinensis* dCCM 7228^T^
Spore position	C S T	C S T	C S	T	T
Temperature(°C)	10, 45	4–45	20–45	15–50	4–28
Oxidase	+	+	+	−	+
D‐Mannitol	−	−	+	+	−

C, Central or paracentral; S, subterminal; T, terminal.

a Data from Yoon and Oh (2005)

b Data from Mehrshad et al. (2013)

c Data from Kwon et al. (2007)

d Data from Wieser, Worliczek, Kampfer, and Busse (2005)

### 3.2 Characterization of alginate lyase secreted by M3

**Figure 2:**
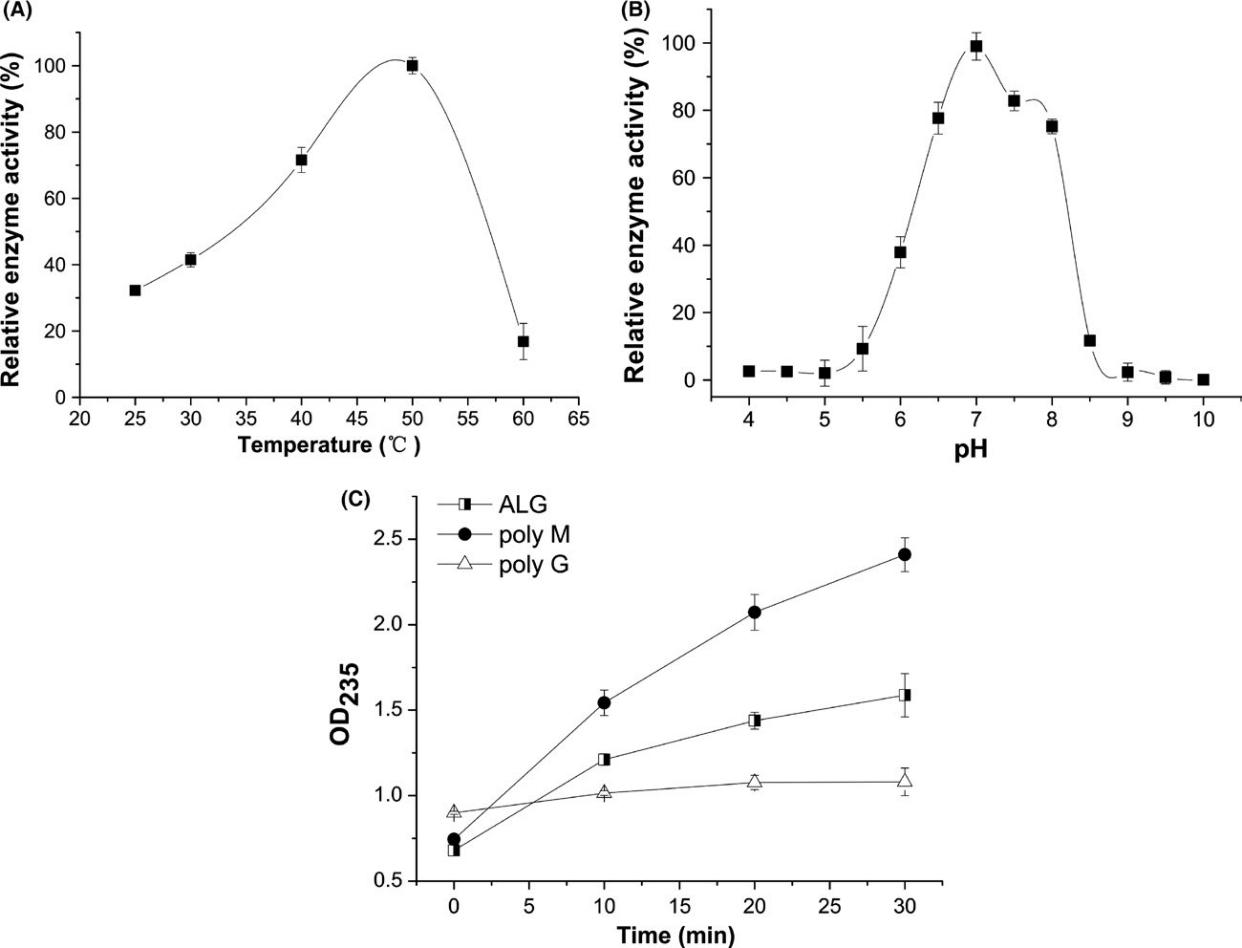
Enzymatic characterization of crude alginate lyase of M3. (A) The optimal temperature of crude enzyme. Reactions were conducted in 50 mM Tris–HCl buffer (pH 7.0) at different temperatures for 10 min. Activity at 50°C was taken as 100%. (B) The optimal pH of crude enzyme. Reactions were conducted at 50°C for 10 min in different buffers over a pH range from 4.0 to 11.0. Activity at pH 7.0 was taken as 100%. (C) Substrate specificity of crude enzyme. Reactions were conducted at 50°C in a mixture consisting of 0.3 ml of crude enzyme and 2.7 ml of the following various substrates in 50 mM Tris–HCl buffer (pH 7.0), crude enzyme, and the following various substrates (0.5%, w/v): sodium alginate (closed squares), poly‐β‐D‐mannuronate (closed circles), poly‐α‐L‐guluronate (closed triangles). The unsaturated uronic acids released were measured by recording the absorbance at 235 nm. Each value represents the mean of three replicates ± standard deviation

The alginate lyase activity of crude enzyme was increased to 33.74 U/mg, compared to 1.32 U/mg in initial fermentation supernatant (Table 2). Overall, a 25‐fold purification was achieved with 47.56% yield in a single step of ammonium sulfate precipitation. The crude enzyme was further characterized biochemically. The enzyme showed maximum activity at 50°C (Fig. 2A). The optimal pH for the enzyme activity was 7.0 (Fig. 2B). Three kinds of substrates were applied to investigate the substrate specificity of crude enzyme (Fig. 2C). The alginate lyase showed higher activity toward polyM than that to alginate and polyG. The activities for polyM were two times higher than polyG. The result indicated that crude enzyme preferred to depolymerize polyM and it might be a member of polyM lyase.

**Table 2:** Enrichment of alginate lyase of M3 strain

Sample	Total protein(mg)	Volume(ml)	Total activity(U)	Specific activity(U/mg)	Purification(fold)	Yield (%)
Fermentation broth	61.8	200	81.58	1.32	1.00	100
Crude enzyme	1.15	1	38.80	33.74	25.56	47.56

### 3.3 Fermentation of *Sargassum horneri*

**Figure 3:**
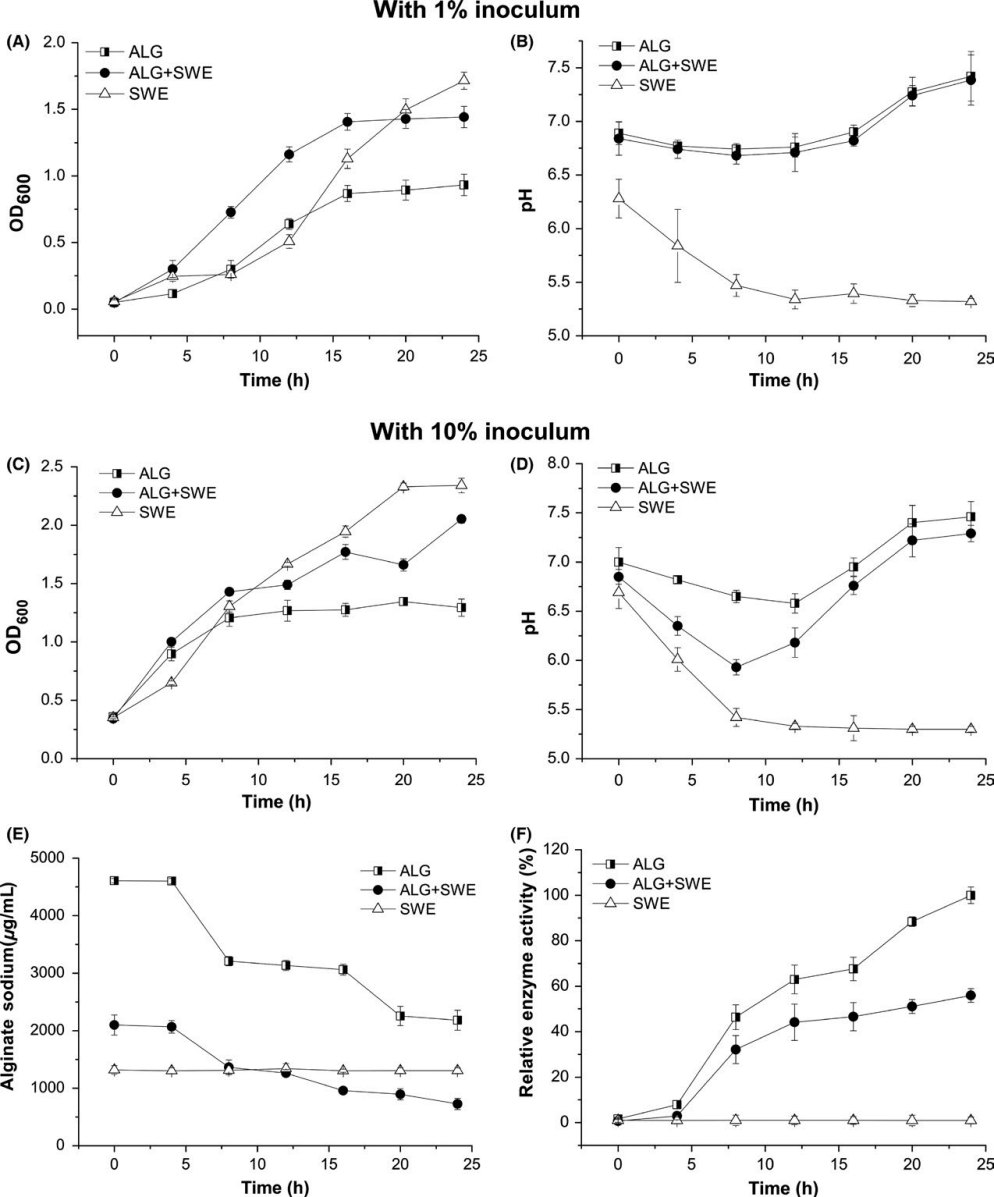
Fermentation characteristics of M3 in different media. (A) The growth curves of M3 in three media with 1% inoculums. (B) The pH curves of M3 in three media with 1% inoculums. (C) The growth curves of M3 in three media with 10% inoculums. (D) The pH curves of M3 in three media with 10% inoculums. (E) Alginate content in three media with 10% inoculums. (F) Alginate lyase activity of fermentation broth in three media with 10% inoculum. An aliquot of 3 ml was drawn at regular interval of 4 hr and analyzed for cell density, pH, soluble alginate content and alginate lyase activity. Three media were ALG (half squares), ALG +  seaweed water extract (SWE) (solid circles), SWE (open triangles), respectively. Each value represents the mean of three replicates ± standard deviation

Shake‐flask fermentation of three different kinds of media with 1% and 10% (v/v) initial inoculums was carried out to identify growth and kinetics of M3 strain and its fermentation products. As shown in Fig. 3A, the growth curves of M3 strain in ALG medium and ALG + SWE medium showed similar change tendency, although the growth rate and biomass in ALG + SWE medium was higher than that in ALG medium. The growth curves also showed that 0–4 hr was lag phase, 4–12 hr was logarithmic phase and after 12 hr was stationary phase in both media. In addition, the pH curves in two kinds of media were almost identical. The pH value decreased to 6.7 during logarithmic phase but increased to 7.4 at stationary phase (Fig. 3B). While the fermentation characteristics are similar between ALG and ALG + SWE media, obvious differences was observed in SWE medium. The logarithmic growth of M3 in SWE medium began at 8 hr, four hours later than that in other two media. The pH was reduced to 5.3 and remained low.

When the initial inoculum was increased to 10% (v/v), the growth and kinetics of M3 were generally similar to the fermentation result of 1% inoculum (Fig. 3C and D). But the logarithmic growth of M3 was four hours earlier than that with 1% inoculum in all three media (Fig. 3A and C). Alginate content and alginate lyase activity were also followed during the fermentation process. In ALG and ALG + SWE media, the amount of alginate decreased about 50% during the growth period (Fig. 3E). The first decrease appeared at the beginning of logarithmic growth and second decrease appeared at stationary phase. This result was related to the increasing tendency of enzyme activity in fermentation broth (Fig. 3F). However, the amount of soluble alginate and enzyme activity in SWE medium remained unchanged.

Our fermentation results indicated that M3 strain secreted alginate lyase into fermentation broth in ALG and ALG + SWE media. Seaweed broth appeared to be promising as a growth medium for M3 strain. The culture had the ability to utilize sugars released from seaweeds.. However, alginate could not be degraded by M3 in seaweed broth. Addition of alginate into seaweed extract might induce M3 to secrete alginate lyase via adjusting the pH of fermentation broth, and this possibility was tested below.

### 3.4 Detection of alginate oligosaccharides

**Figure 4:**
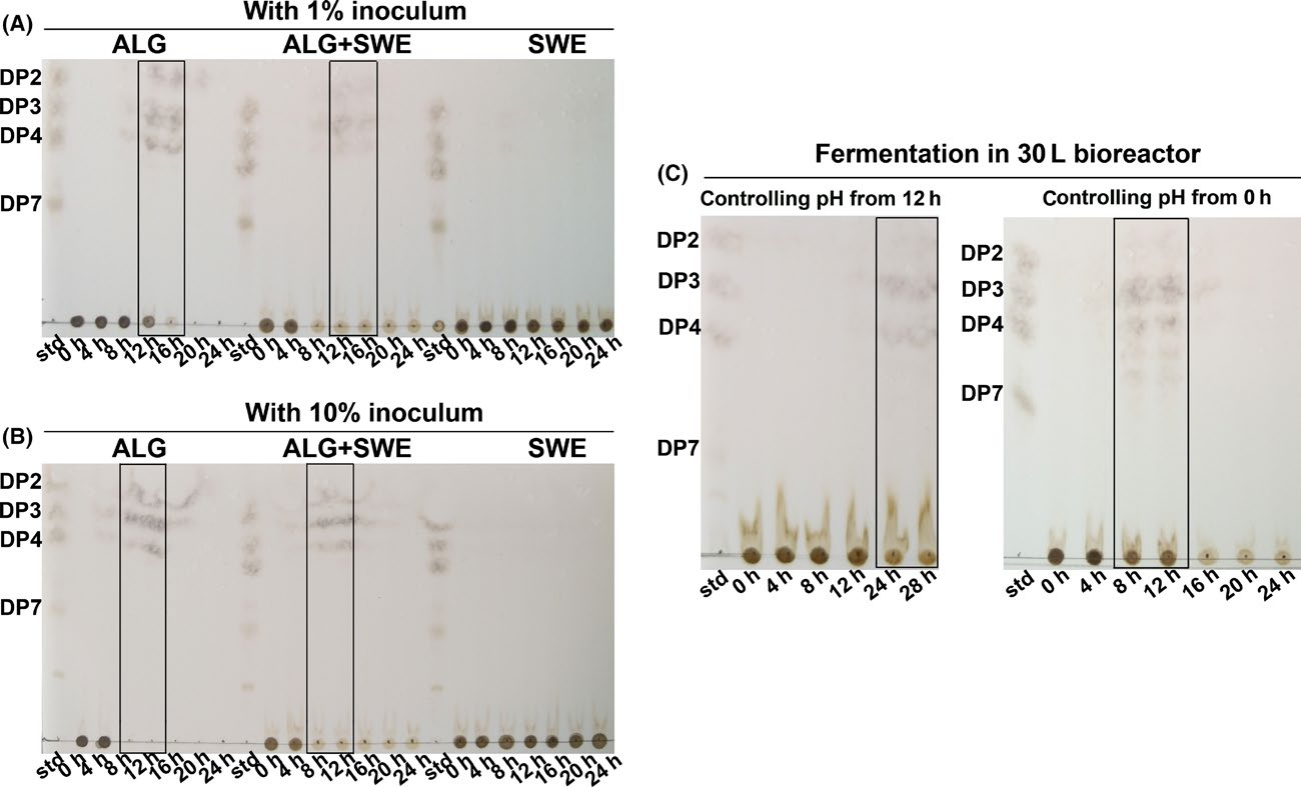
Thin layer chromatography (TLC) analysis of alginate oligosaccharides in fermentation broth. (A) Samples from different fermentation time in three media with 1% inoculums. (B) Samples from different fermentation time in three media with 10% inoculums. (C) Samples from different fermentation time in seaweed water extract (SWE) medium with controlling pH at 7.0. Aliquot samples were taken at the interval of 4 hr and then spotted on the TLC plate. Std: alginate oligosaccharide standards. DP2‐DP7: the mannuronic acid sodium salt dimer, trimer, tetramer, and heptamer. Three media were ALG medium, ALG + SWE medium, and SWE medium, respectively. Rectangles showed the times with maximum concentration of alginate oligosaccharides in different media

In fermentation broth, we found the amount of alginate decreased during fermentation. Alginate could be degraded into oligosaccharides such as homo polymeric G block, M block, alternating MG (GM) block by alginate lyases (Gacesa, 1988; Ji, 1997; Preiss & Ashwell, 1962). Alginate oligosaccharides were the most direct and strong evidence of alginate degradation by alginate lyase. Thin layer chromatography (TLC) was used to detect alginate oligosaccharides with different degrees of polymerization (DP). As shown in Fig. 4, different DP (between 2 and 4) of alginate oligosaccharides was detected in ALG and ALG + SWE fermentation broth, but in SWE medium there were no alginate oligosaccharides. With 1% inoculum (Fig. 4A), alginate oligosaccharides were first detected at 8 hr and were maximized during 12–16 hr. After 16 hr, both DP and the amount of oligosaccharides decreased. At 24 hr, no alginate oligosaccharides could be detected on TLC plate. Associating with the growth curve, we inferred that alginate oligosaccharides might be utilized as carbon source by bacteria. With 10% inoculum (Fig. 4B), alginate oligosaccharides appeared at 4 hr and kept maximum amount during 8–12 hr, which were four hours earlier than that with 1% inoculum. This result suggests that increasing inoculum size could accelerate alginate‐degrading in fermentation and the period of 8–12 hr was the best harvest time to obtain alginate oligosaccharides.

Shake‐flask fermentation results showed that in SWE fermentation broth, pH was a discrimination factor compared with the other two media (Fig. 3B and D). Associating with the result of optimal pH of crude enzyme, we inferred that variation of pH in fermentation broth might affect secretion of alginate lyase or its activity. In order to maintain the pH of fermentation process, a 30 L bioreactor was applied to proceed used for the fermentation with controlling pH at 7.0. Two batch fermentations were carried out with controlling pH from the beginning of fermentation and 12 hr after the inoculation, respectively. As shown in Fig. 4C, alginate oligosaccharides were detected at 24 hr and 28 hr with controlling pH from 12 hr. In the condition of controlling pH during the whole fermentation process, alginate oligosaccharides were detected at 8 hr–12 hr and decreased at 16 hr (Fig. 4C). At 24 hr, no alginate oligosaccharides could be detected on TLC plate. This variation tendency was coincident with that in ALG and ALG + SWE shake‐flask fermentation. This result indicated that the pH was an important factor affecting production of alginate oligosaccharide in seaweed fermentation by M3 strain.

### 3.5 Quantification of alginate oligosaccharide by HPLC

**Figure 5:**
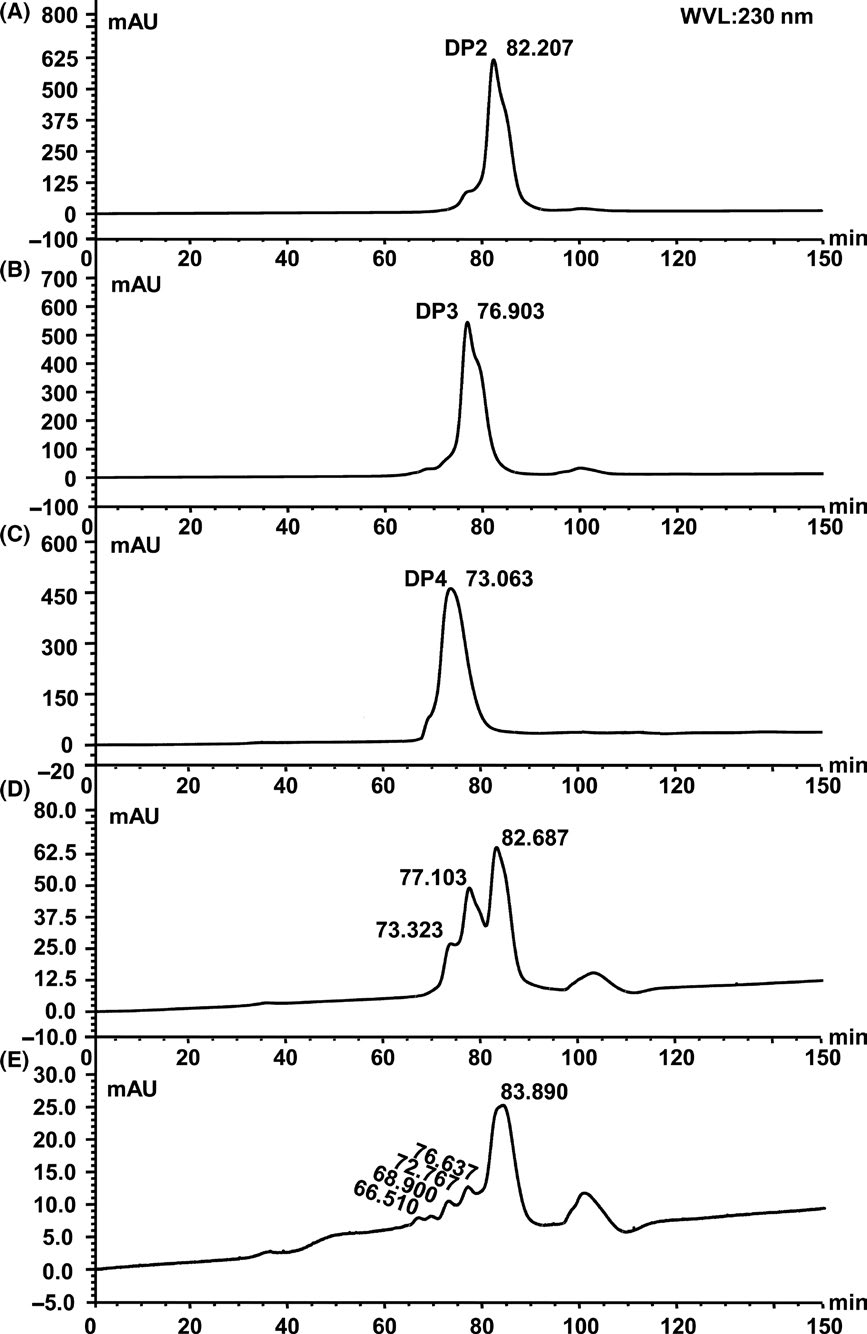
Elution profile of alginate oligosaccharides by HPLC. (A) Mannuronic acid sodium salt dimer. (B) Mannuronic acid sodium salt trimer. (C) Mannuronic acid sodium salt tetramer. (D) Enzymatic hydrolysate solution at 24 hr. (E) Seaweed fermentation broth at 12 hr with controlling pH. An HPLC analysis was carried out with an UltiMate 3,000 system under the following analytical condition: column, Superdex^™^ 30, 10 mm × 300 mm (GE Healthcare); mobile phase, 0.1 mol/L NaCl; column temperature, 25°C; flow rate, 0.2 ml/min; injection volume, 20 μl; monitoring wavelength, 230 nm

To further confirm the presence of alginate oligosaccharide in fermentation broth, HPLC was applied. The chromatogram of alginate oligosaccharide standard was shown in Figure 5A, B and C. The retention times of dimer, trimer, and tetramer were 82.2 min, 76.9 min, and 73.1 min, respectively. As shown in Figure 5D, the peaks corresponding to the dimer, trimer, and tetramer of standard alginate oligosaccharide were detected in enzymatic hydrolysis solution of crude enzyme at 24 hr. The results indicated that the main production of our alginate lyase was oligosaccharide with low DP (2–4). In addition to oligosaccharides with DP 2‐4, we even detected pentamer and hexamer in seaweed fermentation broth with controlling pH, though the concentration of them was very low (Fig. 5E). On the basis of the peak area, we calculated the concentration of oligosaccharides and relative percentage content of each component. In enzymatic hydrolysate at 24 hr, the concentration of alginate oligosaccharide was 1.89 mg/ml and the relative percent contents of oligomers of DP 2, 3, and 4 were 52.5%, 35.5%, and 12%, respectively. In seaweed fermentation broth for 12 hr, the concentration of alginate oligosaccharide was 0.53 mg/ml and the relative percent contents of oligomers of DP 2, 3, 4, 5, and 6 were 73.9%, 14.5%, 7.6%, 2.3%, and 1.6%, respectively.

### 3.6 Seaweed extract promotes plant growth

In order to confirm that alginate oligosaccharides were the main factor in seaweed fermentation broth for promoting plant growth, we collected seaweed fermentation broth containing alginate oligosaccharides at 12 hr (SFE‐12) and at 24 hr (SFE‐24 in which no oligosaccharides were detected), and performed plant growth trials. Alginate oligosaccharides in enzymatic hydrolysis (AOS) were diluted to the same concentration as in SFE‐12 and used as a positive control.

**Figure 6:**
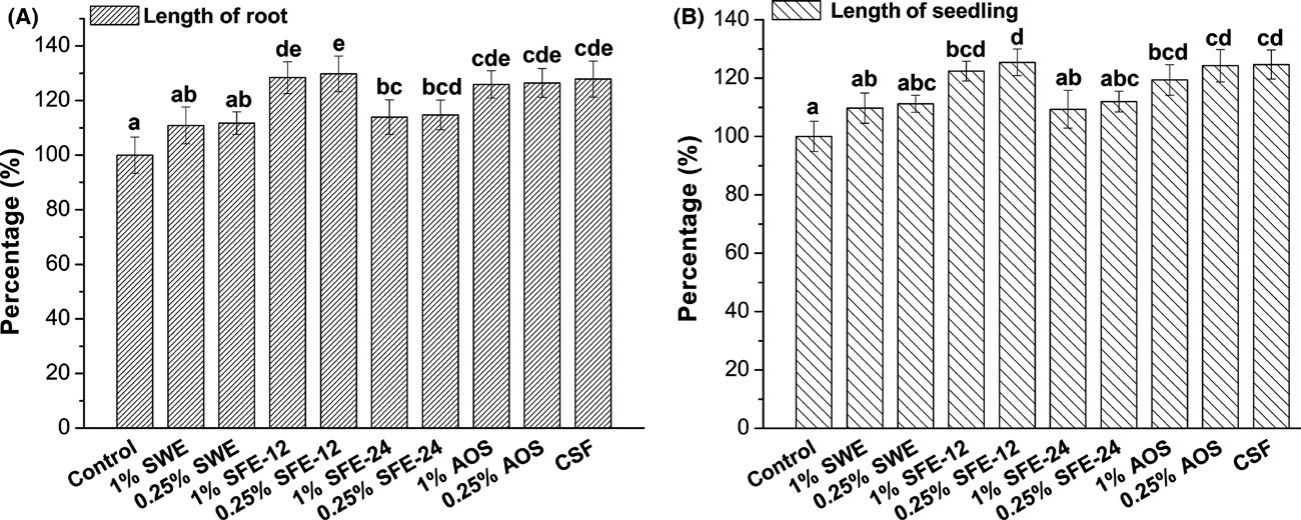
Tomato root and seedling growth promotion effect of different seaweed extract. (A) Root length elongation percentage with different treatments. (B) Seedling length elongation percentage with different treatments. Control: distilled water; seaweed water extract (SWE): seaweed water extract; SFE‐12: seaweed fermentation extract at 12 hr (at constant pH 7); SFE‐24: seaweed fermentation extract at 24 hr (at constant pH 7); AOS: Enzymatic hydrolysate of alginate. CSF: commercial seaweed fertilizer. Error bars represent standard deviation (*n* = 3). Mean values (*n* = 3) with different letters are significantly different (*p *<* *.05) with Duncan's multiple range test (For example, there is a significant difference between “e” and “bcd”, but there is not a significant difference between “e” and “de”). Control was expressed as 100%

In comparison with the control, all doses of seaweed fermentation extracts (SFE‐12 and SFE‐24) significantly promoted the growth of tomato (*Lycopersicon eseulentum* L.) (Fig. 6). Different seaweed extract showed various promotion effects. SFE‐12 showed much better promotion effect for root growth than SWE or SFE‐24 in condition of same diluted concentration (*p *<* *.05). The increased elongation ratio of root (25%–26%) and seedling (19%–24%) in AOS groups was similar to SFE‐12 (root elongated 28%–29%, seedling elongated 22%–25%, respectively). After 7 days growth, the length of root and seedling had a maximum response at the 0.25% concentrations of SFE‐12, 29% and 25% longer than control group, respectively. The promotion effect of the lower dose SFE‐12 (0.25%) was higher than that of the higher dose SFE‐12 (1%), or the commercial seaweed fertilizer. However, this differences were not statistically significant. These results indicated that alginate oligosaccharides promoted the growth of tomato roots and seedling as an important production of seaweed fermentation and the low concentration (0.25%) of seaweed fermentation extract showed better growth‐promoting effect of tomato.

## 4 Discussion

### 4.1 A new alginate lyase discovered in our study

According to reports, the genera of *Pseudomonas* (*Pseudomonas* sp. QD03, *Pseudomonas* sp. F6, *Pseudomonas fluorescens*,* Pseudomonas aeruginose* CF1/M1, *Pseudomonas* sp. Os‐ALG‐9, *Pseudomonas syringae*,* Pseudomonas* sp. E03) (Bakkevig et al., 2005; Kraiwattanapong, Motomura, Ooi, & Kinoshita, 1999; Li, Guan, Jiang, & Hao, 2011; Miyazaki, Obata, Iwamoto, Oda, & Muramatsu, 2001; Preston, Wong, Bender, & Schiller, 2000; Xiao, Han, Yang, Lu, & Yu, 2006) and *Vibrio* (*Vibrio* sp. 510‐64, *Vibrio* sp.W13, *Vibrio alginolyticus*,* Vibrio* sp.YKW‐34) (Deng, Ye, Xu, & Zhang, 2014; Fu, Lin, & Kim, 2007; Hu, Jiang, & Hwang, 2006; Zhu et al., 2015) have shown good ability in producing alginate lyase. But few reports were found from the genus *Bacillus* (Nakagawa et al., 1998). In our study, a fermentative strain M3 was isolated from decayed kelp and identified as *Bacillus litoralis* M3. It was confirmed that M3 strain had the ability to secret alginate lyase to degrade alginate due to degradation of soluble alginate and production of alginate oligosaccharides (Figs. 3E, 4). The polyM‐specific alginate lyase in this study showed optimal temperature of 50°C (Fig. 2A), whereas the optimal temperature of most alginate lyase is below 45°C (Li et al., 2011). Our study suggests that the M3 lyase is a new and unique member in alginate lyase family due to its higher optimal temperature and substrate specificity.

### 4.2 Fermentation of seaweed extract by M3 strain

Fermentation of seaweed was usually applied for bioethanol and bioactivity production (Adams, Gallagher, & Donnison, 2008; Jang, Cho, Jeong, & Kim, 2012; Lee & Lee, 2011). Most seaweed fermentation studies utilized traditional fermentation strains such as yeast, lactic acid bacteria, or mixed microbe (Eom et al., 2011; Shobharani, Halami, & Sachindra, 2012; Uchida & Murata, 2004). In our study, we screened an alginate‐degrading strain for fermentation. The results of fermentation and TLC showed that the M3 strain could secrete alginate lyase to degrade soluble alginate and then produced oligosaccharides in medium of alginate as the sole carbon source, but not in seaweed extract medium. Alginate oligosaccharide was detected after adding alginate to seaweed extract medium (ALG + SWE). Next, we showed that M3 strain could ferment seaweed extract and produce alginate oligosaccharides by controlling pH of fermentation broth. This result confirmed that it was the pH, not the alginate concentration that affected alginate oligosaccharides production.

In addition, we showed that the fermentation of M3 strain is a dynamic process, especially between 8–12 hr, many fermentation factors changed such as pH decreasing, logarithmic growth of bacteria, alginate‐degrading, and production of alginate oligosaccharides (Figs. 3, 4). The kinetics of the fermentation in shake‐flask, 30 L bioreactor and 2000 L bioreactor (Fig. S2) were basically stable due to the consistent time (8–12 hr) for production of alginate oligosaccharides in different scales of fermentations. This study may provide a useful reference for future studies and a useful guide for industrial production of fermented seaweed fertilizer.

### 4.3 Alginate oligosaccharides promoted the plant growth as an effective component in fermentation broth

In plant growth trials, the fermentation extract from *Sargassum horneri* by M3 (SFE‐12) had obvious enhancement effect on root and seedling growth. Its beneficial effect was significantly better than SWE and SFE‐24 (*p *<* *.05) but almost equal to the alginate oligosaccharides solution (AOS). The major difference between fermentation broth SFE‐12 and SFE‐24 was the alginate oligosaccharides. It suggested that fermentation produced alginate oligosaccharides and these oligomers enhanced the promotion function of seaweed extract. Even though many unidentified matters still existed in seaweed fermentation extract, our results indicated that alginate oligosaccharides at least were an effective component for improving plant growth. The SFE‐12 also showed a slightly better, although not statistically significant, growth promotion effect than the commercial seaweed fertilizer. There are no components of alginate oligosaccharides in the commercial seaweed fertilizer. The major components in commercial seaweed fertilizer are alginate acid, N, P, K, Cu, Zn, and Fe. The seaweed fermentation extracts might be a better option, because it likely has less side‐effect to the soil. In addition, alginate oligosaccharides with low degree of polymerization (DP) show multiple effects for plant growth. It has been reported that low DP alginate oligosaccharides (DP 2‐DP 4) promote root formation and growth in wheat (Zhang et al., 2013) and rice (Zhang et al., 2014). They can also enhance wheat tolerance to drought stress (Liu et al., 2013). Similarly, we showed in this study that alginate oligosaccharides of DP 2‐ DP 4 in fermentation broth promote root growth (Fig. 6). However, the mechanism of growth promotion by SFE‐24 or SWE is not clear, since no alginate oligosaccharides were detected. One possibility is that the SFE‐24 or SWE may have a low level of alginate oligosaccharides that we were unable to detect; the other possibility is that the growth promotion is caused by other unidentified components. Although many of the various chemical components of seaweed extracts and their modes of action remain unknown, our results indicate that producing seaweed fertilizer by fermentation is available and effective, and the fertilizer could be potentially used in agriculture.

## Funding Information

This work was supported by the Science and Technology Development Project of Weihai (2010‐3‐96), the National Key Technology R&D Program of China (2013BAB01B0), the Science and Technology Service Network Initiative of Chinese Academy of Sciences (KFJ‐EW‐STS‐060), the Young Scientists Fund of National Natural Science Foundation of China (41401285), the Open Foundation of State Key Laboratory of Estuarine and Coastal Research (SKLEC‐KF201412), the Public Science and Technology Research Funds Projects of Ocean (201505022), and the Chinese Academy of Sciences Strategic Pilot Project (XDA1102040300).

## Conflict of Interests

The authors declare no conflict of interest.
