# Supplementary figures and images for: Isolation of a novel alginate lyase‐producing Bacillus litoralis strain and its potential to ferment Sargassum horneri for biofertilizer
Source: Microbiologyopen. 2016 Jul 20;5(6):1038–49. doi: 10.1002/mbo3.387 (PMC5221473; doi:10.1002/mbo3.387)

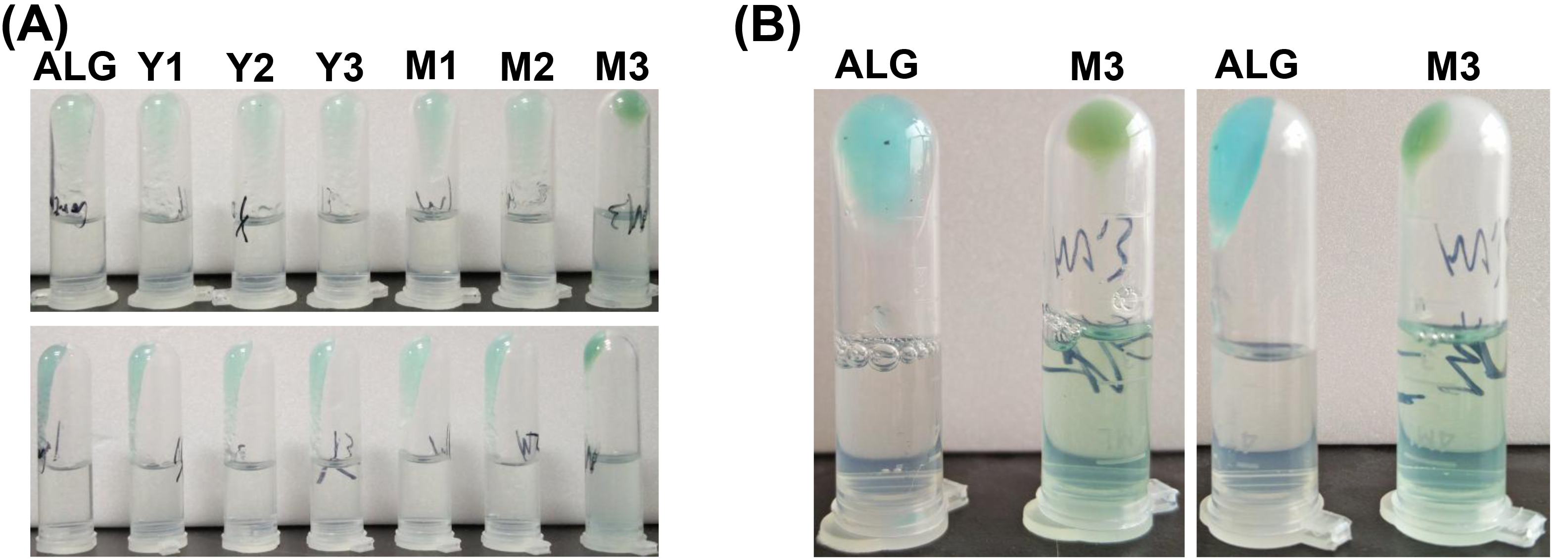

Supplement: Supplementary file 1 [file MBO3-5-1038-s001.tif]

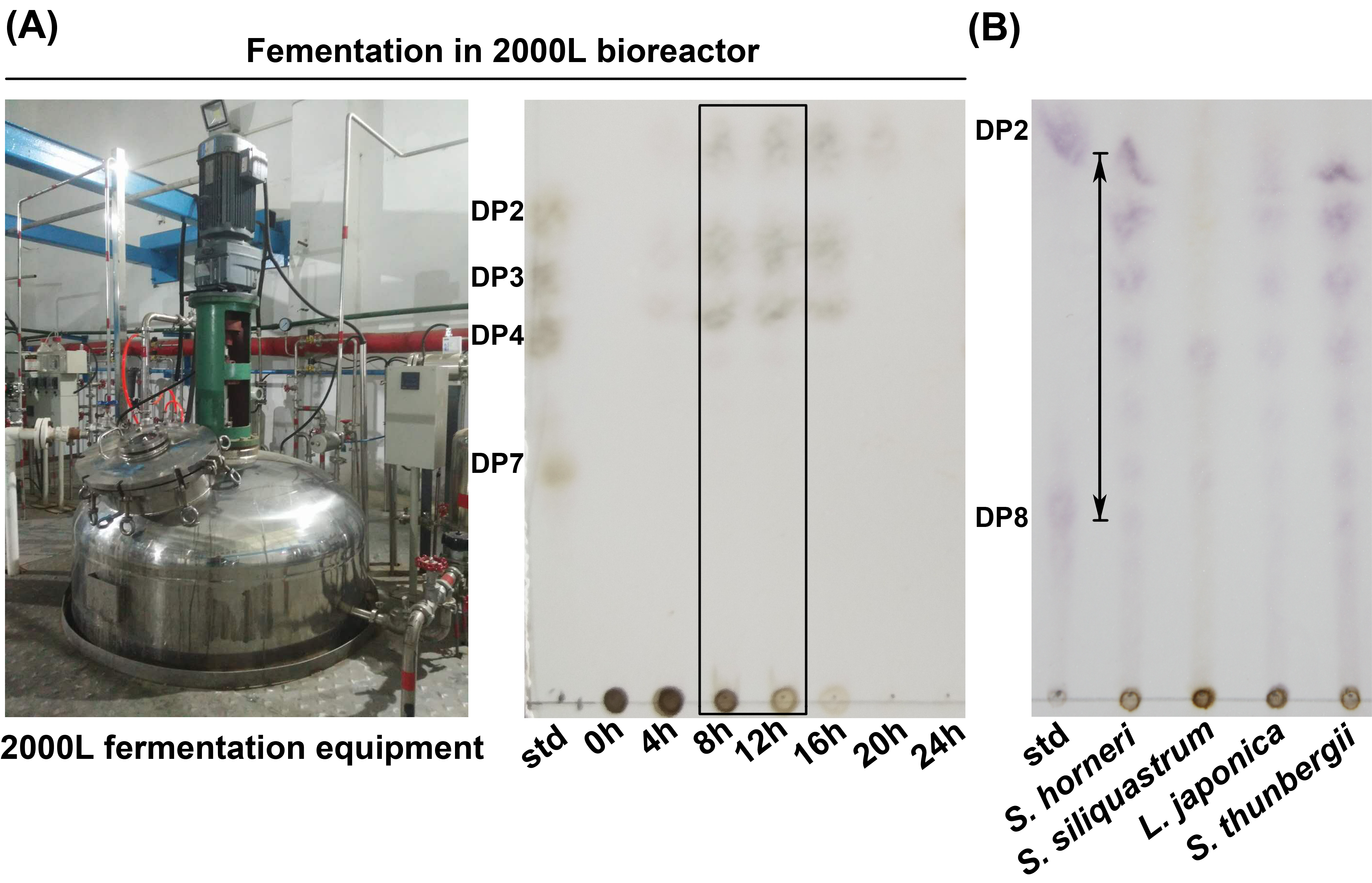

Supplement: Supplementary file 2 [file MBO3-5-1038-s002.tif]
